# Adding value to food chain information: using data on pig welfare and antimicrobial use on-farm to predict meat inspection outcomes

**DOI:** 10.1186/s40813-021-00234-x

**Published:** 2021-10-14

**Authors:** Joana Pessoa, Conor McAloon, Maria Rodrigues da Costa, Edgar García Manzanilla, Tomas Norton, Laura Boyle

**Affiliations:** 1 Pig Development Department, Teagasc Animal and Grassland Research and Innovation Centre, Moorepark, Ireland; 2 Section of Herd Health and Animal Husbandry, School of Veterinary Medicine, University College Dublin, Belfield, Ireland; 3 M3-BIORES-Measure, Model and Manage Bioresponses, KU Leuven, Leuven, Belgium; 4 Epidemiology Research Unit, Department of Veterinary and Animal Science, Northern Faculty, Scotland’s Rural College (SRUC), Inverness, Scotland

**Keywords:** Boosted trees, Food chain information, Machine learning, Meat inspection, Pig, Welfare

## Abstract

**Background:**

Using Food Chain Information data to objectively identify high-risk animals entering abattoirs can represent an important step forward towards improving on-farm animal welfare. We aimed to develop and evaluate the performance of classification models, using Gradient Boosting Machine algorithms that utilise accurate longitudinal on-farm data on pig health and welfare to predict condemnations, pluck lesions and low cold carcass weight at slaughter.

**Results:**

The accuracy of the models was assessed using the area under the receiver operating characteristics (ROC) curve (AUC). The AUC for the prediction models for pneumonia, dorsocaudal pleurisy, cranial pleurisy, pericarditis, partial and total condemnations, and low cold carcass weight varied from 0.54 for pneumonia and 0.67 for low cold carcass weight. For dorsocaudal pleurisy, ear lesions assessed on pigs aged 12 weeks and antimicrobial treatments (AMT) were the most important prediction variables. Similarly, the most important variable for the prediction of cranial pleurisy was the number of AMT. In the case of pericarditis, ear lesions assessed both at week 12 and 14 were the most important variables and accounted for 33% of the Bernoulli loss reduction. For predicting partial and total condemnations, the presence of hernias on week 18 and lameness on week 12 accounted for 27% and 14% of the Bernoulli loss reduction, respectively. Finally, AMT (37%) and ear lesions assessed on week 12 (15%) were the most important variables for predicting pigs with low cold carcass weight.

**Conclusions:**

The findings from our study show that on farm assessments of animal-based welfare outcomes and information on antimicrobial treatments have a modest predictive power in relation to the different meat inspection outcomes assessed. New research following the same group of pigs longitudinally from a larger number of farms supplying different slaughterhouses is required to confirm that on farm assessments can add value to Food Chain Information reports.

## Background

The primary objective of meat inspection (MI) is to identify animals unfit for human consumption. Additionally, MI can also identify animal welfare issues and be used for surveillance and control of animal diseases [1]. MI includes three key elements: food chain information (FCI), *ante mortem* (AM) and *post mortem* (PM) inspections. FCI is the link between farm and slaughterhouse and should provide information regarding pigs’ life that is relevant for meat safety. Recently, official *post mortem* MI in the European Union changed to visual only inspection, thus excluding routine palpation and incision procedures [2]. Indeed, more detailed MI (with palpation/incision techniques) is now only performed in suspect animals, identified through FCI, AM or/and at PM.

However, batches of incoming pigs with a high frequency of health and welfare lesions are not fit for visual MI [3, 4]. These pigs need closer attention from the meat inspector, and palpation and incision procedures are likely also required, which entails a reduction in line speed [5]. Furthermore, although these lesions are not normally associated with foodborne zoonotic agents [6], palpation and incision techniques increase the risk of microbial cross-contamination [6–9].

Ideally, batches of pigs with a high frequency of health and welfare lesions should be slaughtered separately for resources to be allocated efficiently and to ensure microbial meat safety. Thus, information that enables evidence-based risk categorization of slaughter pigs is needed [3]. This could come from two sources: AM findings and/or FCI reports. Nonetheless, considering the practical arrangements needed at the slaughterhouse, basing these measures on AM findings can be impractical and onerous.

According to European Union Regulation (EC) No. 853/2004 [2], FCI should be sent to the slaughterhouse no less than 24 h before the day of slaughter, including information, among others, on animal health status, veterinary products administered within a relevant period and with withdrawal periods greater than zero, and production data that might indicate the presence of disease (i.e. mortality records). Hence, FCI reports could be a more promising way of identifying pigs at risk. Indeed studies show it is possible to incorporate routine on-farm data on animal health (including antimicrobial use—AMU) and welfare information within the FCI [4, 10, 11] in spite of the potential disadvantage of farmers providing insufficient or inaccurate information [4, 11]. Furthermore, there are validated standardized protocols to assess pig health and welfare on farm (Welfare Quality, 2009). These are not done routinely, as they are labour-intensive and time-consuming [13], but there are studies indicating the usefulness of adapted, scaled down versions utilising a few key animal based welfare outcomes [14].

Using FCI data to objectively identify high-risk animals entering abattoirs can represent an important step forward towards improving on-farm animal welfare. Therefore, the aim of this study was to develop and evaluate the performance of a classification model that uses longitudinal on-farm data on pig health and welfare to predict condemnations, pluck lesions and low cold carcass weight at slaughter.

## Methods

This was an observational study whereby pigs were managed according to routine practices on an Irish commercial farm with a wean-to-finish system from July to November 2018. We selected this farm due to its history of respiratory disease, assessed through slaughterhouse checks [15]. This farm was positive for *Mycoplasma hyopneumoniae*, *Actinobacillus pleuropneumoniae*, porcine reproductive and respiratory syndrome virus and Influenza A virus.

Full information regarding animal management is available in [16]. In short, four batches of pigs (n = 1573, in total) of *circa* 12 weeks of age and weighing 25 ± 5.3 kg were housed, on arrival at the farm, in eight rooms each divided into six pens (mean number of pigs: 197 ± 5 per room and 33 ± 2 per pen). All pigs were individually identified with ear-tags and were followed until reaching the target slaughter weight of 110 kg (114 ± 15.4 kg live-weight). Pigs were transferred (at 14 and 18 weeks of age), in the same groups, to the grower and finisher accommodation, respectively. Environmental enrichment was provided by farm staff in the form of hard-plastic balls.

Average daily gain from week 12 to slaughter was 917 ± 45.2 g/day. Feed conversion ratio and finisher mortality in 2018 were 2.52 and 3.9%, respectively. The equivalent figures for the Irish finisher herd were 2.72 and 2.4%, respectively [17].

### On-farm data collection

On arrival at the farm and at transfer between each production stage all pigs were individually weighed and assessed for animal-based welfare outcomes associated with the good housing and health themes of Welfare Quality® (Table 1). The first author carried out all assessments.

**Table.1:** Animal-based welfare outcomes recorded during welfare assessment on a commercial farm ( adapted from van Staaveren et al., 2018)

Welfare criteria	Outcome	Description and scoring procedure
Comfort around lying area	Bursitis	Presence (1) or absence (0) of inflamed bursae on limb(s)
Absence of injuries	Lameness	Presence (1) or absence (0) of claudication
	Tail lesions	Score 0 (no evidence of tail biting) to 4 (total loss of the tail)
	Ear lesions	Score 0 (intact) to 4 (loss of one or both ears)
Absence of disease	Hernia	Umbilical or inguinal hernias: small, medium or large in size^1^

^1^ Subjective classification: small (approximately golf-ball size); medium (approximately baseball size); large (approximately melon size) by [19]

Data on antimicrobial treatments (AMT) were registered daily by farm staff on a per pig basis. Antimicrobials were only administered parenterally during the trial. For each treatment, information on pig ID, date, commercial name of the active substance, dosage, and reason for treatment was recorded. Only the number of antimicrobial treatments per pig was included in the modelling approach.

### Slaughterhouse data collection

Pigs were sent to the slaughterhouse in eight batches, which corresponded to the eight rooms where they were housed on farm. A unique slap number linked to each pig’s ear-tag number was used, thus allowing the attribution of each carcass to the corresponding individual pig. All pluck examinations were carried out by the same trained veterinarian.

Data collection on lung lesions followed the same protocol reported in [16]. Briefly, for each pig, individual lung lobes were examined for pneumonia lesions according to the method developed by Madec & Derrien (1981). The scores were 0 (no pneumonia) to 4 (76–100% of the lung lobe affected). Pleurisy was scored on the dorsocaudal (DC) lobes utilizing a modified version of the Slaughterhouse Pleurisy Evaluation System [21]. The scores were 0 (no pleurisy), 2 (focal lesions in one lobe), 3 (bilateral adhesions or monolateral adhesions affecting more than 1/3 of the diaphragmatic lobe), and 4 (extensive lesions affecting more than 1/3 of both diaphragmatic lobes). Cranial pleurisy (CP) and pericarditis were recorded as absent (0) or present (1).

Tail lesion scoring was performed using the same scoring system applied on-farm (Table 1). In total, three assessors performed this scoring after having received training.

All carcasses diverted for total or partial condemnation, according to the decision of the acting veterinary meat inspector, and reason for condemnation were recorded. Because the presence of cranial pleurisy and the severity of dorsocaudal pleurisy were recorded for all pigs, all carcasses for which the reason for partial condemnation was due to pleural adhesions to the chest wall were not included to calculate the prevalence of partial condemnations. Individual cold carcass weights were also recorded.

### Model building procedure

Six models were built. Slaughterhouse variables were transformed into six binary variables: 1) presence of pneumonia (scores ≥ 1), 2) presence of moderate to severe dorsocaudal pleurisy (scores ≥ 3), 3) presence of cranial pleurisy, 4) presence of pericarditis, 5) partial and total condemnations, and 6) low carcass weight (carcasses belonging to the 15% of animals with lowest cold carcass weight). A second observer had to be drafted to conduct tail lesion scoring for one of the batches of pigs at slaughter resulting in considerable inconsistencies between observers. Therefore, these lesions were not included. On-farm variables were used as predictor variables (Table 1). All animal-based welfare outcomes assessed at week 12, 14 and 18 were used with no transformation. Antimicrobial treatment was used as a continuous variable, indicating the number of treatments each pig received from week 12 until slaughter. Pig was considered the experimental unit.

The Gradient Boosting Machine algorithm (GBM) was used to evaluate the potential of health and welfare variables to predict partial and total condemnations, pluck lesions and low cold carcass weight at slaughter. The GBM algorithm is a machine learning technique, specifically a boosted tree algorithm. GBM fits additive models in a forward, stage-wise manner. At each iteration, GBM identifies “shortcomings”, attributing more weight to instances predicted wrongly in the previous iteration [22].

The gbm R package was used for this analysis [23]. Several default model hyperparameters were changed from default values. The initial number of trees was set to 5000, maximum number of splits per tree was set to 3, and shrinkage was set to 0.001. Cross validation was used, with cv = 10, to determine the optimum number of trees (early stopping) to minimize Bernoulli deviance.

### Assessing model performance

To characterize the overall performance of the models, sensitivity and specificity were calculated at several probability thresholds. Sensitivity was defined as the proportion of pigs presenting pluck lesions, suffering partial and total condemnations, or with low carcass weight (belonging to the 15% lowest cold carcass weight) that were correctly identified as such (true positive rate). Specificity was defined as the proportion of pigs with none of the above that were correctly identified as having no lesions/condemnations/low cold carcass weight (true negative rate).

The accuracy of the model was assessed using the area under the receiver operating characteristics (ROC) curve (AUC). AUC values were considered non-informative (AUC = 0.5), less accurate (0.5 < AUC ≤ 0.7), moderately accurate (0.7 < AUC ≤ 0.9), highly accurate (0.9 < AUC < 1), and perfect (AUC = 1; Greiner et al., 2000).

To test the six models on independent data, our dataset was divided into a training dataset, each time an 80% random sample, and a testing dataset (the remaining 20%).

### Variable importance

GBM models display information on the relative influence of each predictor variable, based on whether a variable was selected to split on during the tree building process, and on the magnitude of the reduction of the loss function (i.e. Bernoulli loss) as a result.

Variable importance was expressed as the percentage contribution of each variable in the prediction of each response variable.

## Results

Overall, 22% of pigs had pneumonia lesions at slaughter. The prevalence of dorsocaudal and cranial pleurisy was 10% and 16%, respectively. Pericarditis was recorded in 18% of pigs. Regarding condemnations, 2.6% and 0.8% of pigs were partially and totally condemned, respectively.

The prevalence of the different animal-based welfare outcomes is presented in Table 2. The average number of parenteral antimicrobial treatments administered on farm was 0.23 (min. 0 and max. 8).

**Table.2:** Percentage frequencies of categorical variables used as input to predict pluck lesions, partial condemnations, and low cold carcass weight

Health and welfare lesions	Presence (1) or Absence (0) and lesion scores				
	0	1	2	3	4
Bursitis W12	95.2	4.8	NA	NA	NA
Bursitis W14	88.5	11.5	NA	NA	NA
Bursitis W18	80.1	19.9	NA	NA	NA
Lameness W12	97.7	2.3	NA	NA	NA
Lameness W14	96.1	3.9	NA	NA	NA
Lameness W18	95.5	4.5	NA	NA	NA
Tail lesions W12	97.4	2.4	0.2	0	0
Tail lesions W14	87.5	10.8	1.7	0	0
Tail lesions W18	88.5	8.7	2.7	0.1	0
Ear lesions W12	60.7	14.5	8.7	16.1	0
Ear lesions W14	71.8	3.2	1.5	23.4	0.1
Ear lesions W18	66.7	0	0	33.3	0
Hernias W12^*^	98.8	0.8/0.3	0.1/0	0/0	NA
Hernias W14^*^	98.2	0.8/0.1	0.4/0.2	0.3/0	NA
Hernias W18^*^	97.1	0.9/0.1	0.9/0.1	0.8/0.1	NA

*NA* not applicable

^*^Umbilical/inguinal hernias: small (1), medium (2), large (3) in size

**Fig. 1:**
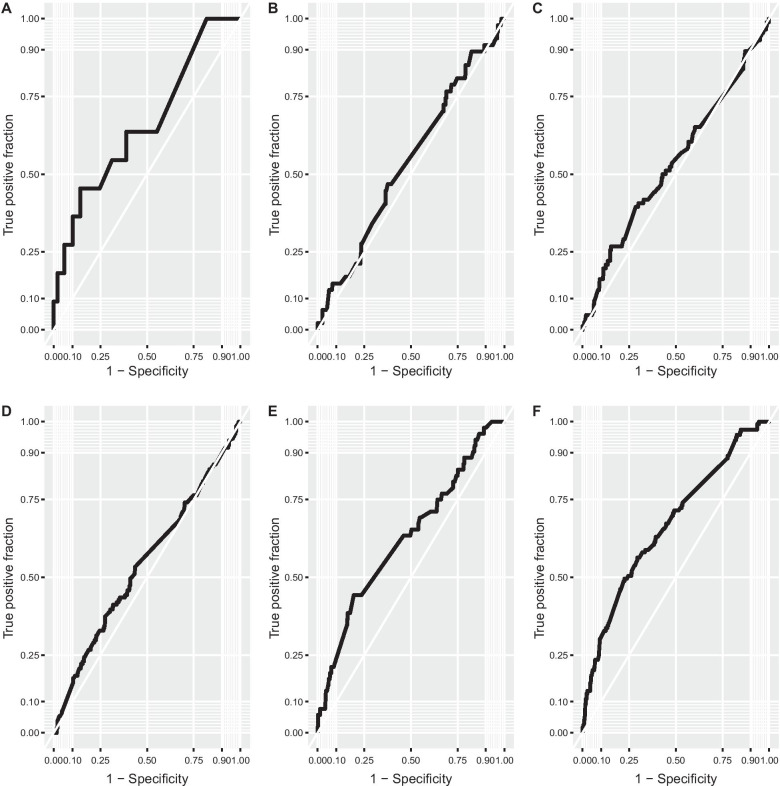
Receiver operating characteristic curves, based on all predicted probabilities of the observations in the test datasets for partial and total condemnations (graph **A**; AUC = 0.66), pneumonia (graph **B**; AUC = 0.54), cranial pleurisy (graph **C**; AUC = 0.60), pericarditis (graph **D**; AUC = 0.60), dorsocaudal pleurisy (graph **E**; AUC = 0.66), and low cold carcass weight (graph **F**; AUC = 0.67)

Table 3 shows the performance of the prediction models for pneumonia, dorsocaudal pleurisy, cranial pleurisy, pericarditis, partial and total condemnations, and low cold carcass weight. Evaluation on the test dataset showed that all models belonged to the less accurate category (0.5 < AUC ≤ 0.7), varying from 0.54 for pneumonia and 0.67 for low cold carcass weight. Furthermore, Fig. 1 shows the ROC curves for each prediction model in the test dataset, where the trade-offs between sensitivity and specificity can be observed.

**Table.3:** Performance characteristics of the boosted tree models: area under the receiver operating characteristic curve (AUC) for six binary slaughterhouse response variables

Predicted variable	Train dataset AUC (95% CI)	Test dataset AUC (95% CI)
Pneumonia	0.62 (0.59–0.66)	0.54 (0.45–0.63)
Dorsocaudal pleurisy	0.66 (0.61–0.71)	0.66 (0.60–0.71)
Cranial pleurisy	0.62 (0.58–0.68)	0.60 (0.54–0.64)
Pericarditis	0.65 (0.61–0.70)	0.60 (0.55–0.63)
Partial and total condemnations	0.72 (0.63–0.80)	0.66 (0.48–0.84)
Low cold carcass weight	0.77 (0.73–0.81)	0.67 (0.61–0.72)

The 10 most important variables per prediction model are shown in Table 4. For predicting pneumonia, which resulted in the weakest performing model, several variables contributed interchangeably. For dorsocaudal pleurisy, ear lesions (assessed on week 12) and AMT were the most important prediction variables. Similarly, the most important variable for the prediction of cranial pleurisy was the number of AMT. In the case of pericarditis, ear lesions (assessed both at week 12 and 14) were the most important variables and accounted for 33% of the Bernoulli loss reduction. For predicting partial and total condemnations, the presence of hernias (on week 18) and lameness (on week 12) accounted for 27% and 14% of the Bernoulli loss reduction, respectively. Finally, AMT (37%) and ear lesions assessed on week 12 (15%) were the most important variables for predicting pigs with low cold carcass weight.

**Table.4:** The 10 most important variables expressed as its percentage (%) contribution in the prediction of each response variable for predicting pigs with pluck lesions, partial and total condemnations, and low cold carcass weight

Variable name	Pneumonia	DC pleurisy	Cranial pleurisy	Pericarditis	Partial and total condemn	Low cold carcass weight
AM treatments	12.5	12.4	20.5	11.7	6.4	37.2
Ear lesions W12	12.2	21.8	15.5	19.6	10.1	14.7
Hernia W18	12.1	4.0	3.9		27.3	1.3
Ear lesions W14	11.6	12.0	9.7	13.4	4.4	13.0
Tail lesions W18	9.1	10.8	11.2	12.9		5.1
Tail lesions W14	8.2	6.4	8.4	7.1	14.0	4.7
Bursitis W14	5.6	4.3	6.2	4.8		7.3
Hernia W14	5.0				5.0	
Bursitis W18	4.5	5.1	7.2	6.5	2.7	7.4
Ear lesions W18	4.0	5.8	5.9	5.9		6.0
Lameness W18		6.7		4.5	6.0	
Lameness W14			2.8	4.3		
Lameness W12					14.2	
Tail lesion W12					3.1	
Bursitis W12						1.3

## Discussion

This study provides results of animal-based welfare outcomes and antimicrobial use assessed on farm and their ability to predict MI outcomes, pluck lesions, and poor performance assessed at slaughter. The prevalence of all animal-based welfare outcomes included in this study, with the exception of tail lesions, was higher compared to the averages for pigs in the different production stages across 31 Irish farms [18]. Regarding the prevalence of pluck lesions, pneumonia and its severity was higher, while dorsocaudal and cranial pleurisy were similar compared to recent findings of an Irish cross sectional study [15]. Cold carcass weight was higher than the average for the Irish pig herd in 2018, while average daily gain was comparable [17]. Regarding partial and total condemnations, our findings were higher compared to a large scale observational study carried out in slaughterhouses in the Republic and Northern Ireland [25]. Although only one farm and one slaughterhouse participated in the study, and thus results should not be extrapolated or interpreted as general figures, we would like to highlight that the same group of individually identified pigs was followed longitudinally from farm to slaughter. Although several studies present results providing associations between animal-based welfare outcomes assessed on farm and different variables assessed at the slaughterhouse [4, 26, 27], all acknowledged that not assessing the same pigs on farm and at slaughter was a major limitation in their methodology. Furthermore, we would like to highlight that differences in farms’ health status, prevalence of specific animal-based welfare outcomes, and performance could possibly influence the different variables assessed at slaughter.

Overall, ear lesions were the most prevalent animal-based welfare outcome scored during all assessments carried out on farm, with the prevalence varying from 33 to 39% (at 18 weeks of age and at 12 weeks, respectively). These findings are considerably higher than average figures reported in studies carried out in Denmark, Chile, and Spain [26, 28, 29]. These studies reported bursitis as one of the most prevalent animal-based welfare outcomes, which is in accordance with our findings (second most prevalent outcome).

Differences in scoring conditions may be the reason for different animal-based welfare outcomes reported in some studies as, in these studies, assessments were conducted on group level and from outside the pens [18, 26, 28, 29]. In the current study, pigs were scored individually, outside the home pen, and with good light conditions. This likely led to more accurate recording of the welfare lesions. Even though it would be too onerous to collect such data in this way under practical conditions, Precision Livestock Farming tools could be employed [30]. Furthermore, although it is possible to collect information on animal-based welfare outcomes at slaughter during MI, several studies expressed concerns regarding its feasibility both due to time constraints and overcrowding [26, 31, 32].

Regarding our modelling approach, machine-learning techniques, as is the case of the GBM models used in this study, have become more commonly used in the animal science field [33]. These techniques are able to deal with a wide range of data types, incomplete datasets, collinear variables, and other assumptions that need to be met by classical regression techniques [34]. Moreover, these models can equal or outperform logistic regression models [33].

Overall, all models run with the test dataset were less accurate (0.5 < AUC ≤ 0.7), with the prediction model for pneumonia having the poorest performance (AUC = 0.54). Indeed, other studies showed that coughing may be the best on-farm indicator to predict pneumonia lesions at slaughter [16]. Dorsocaudal pleurisy, with an AUC of 0.66, was the second best performing model. The most valuable variables to predict these pluck lesions were ear lesions assessed on week 12 and the number of AMT administered. Similarly, the prediction model for cranial pleurisy (AUC = 0.60) used the same variables to obtain the biggest reduction of Bernoulli loss, but in the reverse order. Although medication was administered by farm staff, we collected information on the reasons for its use, and indeed most AMs were administered when farm staff identified pigs with clinical signs of respiratory disease. Still, other studies found that the presence of pleurisy at slaughter was associated with farmers reporting, through the FCI, the presence of coughing for the last 3 months of the rearing cycle [4, 10]. However, we have previously identified strong associations between the presence of coughing and a higher prevalence of different lung lesions at slaughter, but only on the last weeks of the finisher stage in the same farm [16]. Nevertheless, coughing modelling was done at pen and room level in that study. Here, we report results at pig level. Due to the different approaches and related implications (i.e. coughing at pig level would be difficult to report in a commercial setting), we cannot directly compare these studies. Still, these results indicate that it may be useful to include, in the FCI, information on medication use and on different animal-based welfare outcomes, other than coughing, assessed throughout the finisher stage, in order to predict incoming pigs with high prevalence of pleurisy lesions.

The prediction model for pericarditis (AUC = 0.60) showed that ear lesions scored during the three on-farm assessments accounted for 39% of the Bernoulli loss (Table 4), with the first two assessments being the most important variables to predict pericarditis at slaughter. Pericarditis is a common lesion seen at MI across the world [15, 35, 36]. Due to the chronic nature of these lesions, there is uncertainty regarding its etiology [15]. Pathogens such as *Mycoplasma hyopneumoniae, Glaesserella parasuis*, and *Streptococcus suis* are associated with pericarditis [37]. Of these pathogens, *S. suis* is the only one that has ear biting as an associated risk factor [38]. Furthermore, a recent study showed that pig saliva is the major natural habitat of *S. suis,* suggesting that saliva is the most probable source of infection by *S. suis* [39]. To our knowledge, this is the first study where a relationship between pericarditis and ear lesions is reported. In light of these findings, further studies should investigate this association.

The prediction model for partial and total condemnations (AUC = 0.66) showed that the presence of hernias (assessed on week 18) and lameness (assessed on week 12) were the most important predictive variables. These results are in line with the most prevalent reasons for both partial (arthritis) and total condemnations (peritonitis and presence of hernias) in our study, as per the decision of the official veterinarian. Felin et al. [10] also found associations between the presence of hernias and total condemnations.

The prediction model for low cold carcass weight had the best performance of all models (AUC = 0.67). The most important predictive variable was the administration of antimicrobials. This model was included due to the importance of cold carcass weight to farmers (as a proxy for production efficiency), and because meat inspectors are more likely to pay closer attention to smaller/lighter pigs during MI. Although the performance of this model is not high, these results indicate that pigs that required more AMT had poorer growth. Excessive use and misuse of antimicrobials is associated with inappropriate practices, such as improper dosage and dilutions, and insufficient treatment time when using parenteral antimicrobials [40]. More studies are needed to understand if it is more profitable to raise pigs that require long treatment time up to slaughter, or to euthanize them. On the other hand, these pigs may pose a higher food safety risk than non-medicated pigs due to increased risk for the presence of antimicrobial residues and potential risk for antimicrobial resistance. Therefore, it may be useful to individually identify pigs that required AMT during finisher stage and include these details in the FCI, both so farmers can track how many AMT are administered during the whole period, and so that risk categorization of these pigs may be performed at the slaughterhouse.

Interestingly, predictive variables recorded earlier in the finisher stage often scored as the most important variables in several prediction models (Table 4). Normally, lesions that occur later in the finisher stage are better predictors of lesions occurring at slaughter, especially in the case of respiratory diseases [41]. These results show the importance of following pigs longitudinally, starting earlier in the growing/finisher stage.

## Conclusion

The findings from our study show that on farm assessments of animal-based welfare outcomes and information on antimicrobial treatments have a modest predictive power in relation to the different meat inspection outcomes assessed. New research following the same group of pigs longitudinally from a larger number of farms, with different health status and prevalence of animal-based welfare outcomes, and supplying different slaughterhouses is required to confirm that on farm assessments can add value to Food Chain Information reports.

## Data Availability

The datasets used and/or analyzed during the current study are available from the corresponding author on reasonable request.

## Abbreviations

MI: Meat inspection
FCI: Food chain information
AM: Ante mortem
PM: Post mortem
AMU: Antimicrobial use
DC: Dorsocaudal pleurisy
CP: Cranial pleurisy
GBM: Gradient boosting machine
ROC: Receiver operating characteristics curve
AUC: Area under the receiver operating characteristics curve
NA: Not applicable
AMT: Antimicrobial treatments

## References

[1] Stärk KDC, Alonso S, Dadios N, Dupuy C, Ellerbroek L, Georgiev M (2014). Strengths and weaknesses of meat inspection as a contribution to animal health and welfare surveillance. Food Control.

[2] European Council. Regulation (EC) No. 854/2004 of the European Parliament and of the Council of 29 April 2004 laying down specific rules for the organisation of official controls on products of animal origin intended for human consumption. 2004.

[3] Buncic S, Alban L, Blagojevic B (2019). From traditional meat inspection to development of meat safety assurance programs in pig abattoirs—The European situation. Food Control.

[4] Felin E, Hälli O, Heinonen M, Jukola E, Fredriksson-Ahomaa M (2018). Assessment of the feasibility of serological monitoring and on-farm information about health status for the future meat inspection of fattening pigs. Prev Vet Med.

[5] Laukkanen-Ninios R, Rahkila R, Oivanen L, Wirta ER, Fredriksson-Ahomaa M (2020). Views of veterinarians and meat inspectors concerning the practical application of visual meat inspection on domestic pigs in Finland. J fur Verbraucherschutz und Leb..

[6] EFSA. Scientific Opinion on the public health hazards to be covered by inspection of meat (swine). EFSA J. 2011. 10.2903/j.efsa.2011.2351 10.2903/j.efsa.2013.3265 PMC7163758 32313569

[7] Delhalle L, De Sadeleer L, Bollaerts K, Farnir F, Saegerman C, Korsak N (2008). Risk Factors for Salmonella and Hygiene Indicators in the 10 Largest Belgian Pig Slaughterhouses. J Food Prot.

[8] Nesbakken T, Eckner K, Høidal HK, Røtterud O-J (2003). Occurrence of *Yersinia enterocolitica* and *Campylobacter* spp. in slaughter pigs and consequences for meat inspection, slaughtering, and dressing procedures. Int J Food Microbiol.

[9] Pointon AM, Hamilton D, Kolega V, Hathaway S (2000). Risk assessment of organoleptic postmortem inspection procedures for pigs. Vet Rec.

[10] Felin E, Jukola E, Raulo S, Heinonen J, Fredriksson-Ahomaa M (2016). Current food chain information provides insufficient information for modern meat inspection of pigs. Prev Vet Med.

[11] van Wagenberg CPA, Backus GBC, van der Vorst JGAJ, Urlings BAP (2012). Usefulness of food chain information provided by Dutch finishing pig producers to control antibiotic residues in pork. Prev Vet Med.

[12] Quality Welfare. Welfare Quality ® Assessment protocol for pigs. Welf Qual Assess Protoc Pigs. 2009;1–123.

[13] Dalmau A (2014). Health and Welfare Management of Pigs Based on Slaughter Line Records. J Dairy Vet Anim Res.

[14] Van Staaveren N, Doyle B, Manzanilla EG, Calderón Díaz JA, Hanlon A, Boyle LA (2017). Validation of carcass lesions as indicators for on-farm health and welfare of pigs. J Anim Sci.

[15] Rodrigues da Costa M, Fitzgerald RM, Manzanilla EG, O’Shea H, Moriarty J, McElroy MC (2020). A cross-sectional survey on respiratory disease in a cohort of Irish pig farms. Ir Vet J.

[16] Pessoa J, Rodrigues da Costa M, García Manzanilla E, Norton T, McAloon C, Boyle L (2021). Managing respiratory disease in finisher pigs: Combining quantitative assessments of clinical signs and the prevalence of lung lesions at slaughter. Prev Vet Med.

[17] Teagasc. National Pig Herd Performance Report 2018. [Internet]. 2019 [cited 2021 May 10]. Available from: https://www.teagasc.ie/publications/2019/national-pig-herd-performance-report-2018.php

[18] van Staaveren N, Calderón Díaz JA, Manzanilla EG, Hanlon A, Boyle L (2018). Prevalence of health and welfare issues in the weaner and finisher stages on 31 pig farms. Ir Vet J.

[19] Straw B, Bates R, May G (2009). Anatomical abnormalities in a group of finishing pigs: prevalence and pig performance. J Swine Heal Prod.

[20] Madec F, Derrien M. Fréquence, intensité et localisation des lésions pulmonaires chez le porc charcutier: Résultats d’une premiére série d’observations en abattoir. In: Journées de la Recherche Porcine en France. 1981. p. 231–6.

[21] Dottori M, Nigrelli AD, Merialdi G, Gozio S, Bonilauri P, Cominotti. F. Proposta di un nuovo sistema di punteggiatura delle pleuriti suine in sede di macellazione. La griglia S.P.E.S. (Slaughterhouse Pleuritis Evaluation System). Large Anim Rev. 2007;13:161–5.

[22] Witten IH, Frank E, Hall M a. Data Mining: Practical Machine Learning Tools and Techniques [Internet]. 3rd ed. Elsevier; 2011. Available from: http://books.google.com/books?id=bDtLM8CODsQC&pgis=1

[23] Ridgeway G. gbm: generalized boosted regression models. 2010.

[24] Greiner M, Pfeiffer D, Smith RD (2000). Principles and practical application of the receiver-operating characteristic analysis for diagnostic tests. Prev Vet Med.

[25] Harley S, More S, Boyle L, Connell NO, Hanlon A (2012). Good animal welfare makes economic sense: potential of pig abattoir meat inspection as a welfare surveillance tool. Ir Vet J.

[26] Teixeira DL, Salazar LC, Enriquez-Hidalgo D, Boyle LA (2020). Assessment of Animal-Based Pig Welfare Outcomes on Farm and at the Abattoir: A Case Study. Front Vet Sci.

[27] Maisano AM, Luini M, Vitale N, Rota Nodari S, Scali F, Alborali GL (2020). Animal-based measures on fattening heavy pigs at the slaughterhouse and the association with animal welfare at the farm level: a preliminary study. Animal.

[28] Petersen HH, Nielsen EO, Hassing AG, Ersbøll AK, Nielsen JP (2008). Prevalence of clinical signs of disease in Danish finisher pigs. Vet Rec.

[29] Temple D, Dalmau A, Ruiz De La Torre JL, Manteca X, Velarde A (2011). Application of the Welfare Quality® protocol to assess growing pigs kept under intensive conditions in Spain. J Vet Behav..

[30] Larsen MLV, Wang M, Norton T (2021). Information technologies for welfare monitoring in pigs and their relation to welfare quality®. Sustain.

[31] Jackowiak J, Kiermeie A, Kolega V, Missen G, Reiser D, Pointon A (2006). Assessment of producer conducted antemortem inspection of market pigs in Australia. Aust Vet J.

[32] Petersen HH, Enøe C, Nielsen EO (2004). Observer agreement on pen level prevalence of clinical signs in finishing pigs. Prev Vet Med.

[33] Mollenhorst H, Ducro BJ, De Greef KH, Hulsegge I, Kamphuis C (2019). Boosted trees to predict pneumonia, growth, and meat percentage of growing-finishing pigs. J Anim Sci.

[34] Friedman JH (2001). Greedy function approximation: A gradient boosting machine. Ann Stat.

[35] Nielsen SS, Nielsen GB, Denwood MJ, Haugegaard J, Houe H (2015). Comparison of recording of pericarditis and lung disorders at routine meat inspection with findings at systematic health monitoring in Danish finisher pigs. Acta Vet Scand.

[36] Schleicher C, Scheriau S, Kopacka I, Wanda S, Hofrichter J, Köfer J (2013). Analysis of the variation in meat inspection of pigs using variance partitioning. Prev Vet Med.

[37] Buttenschøn J, Friis NF, Aalbaek B, Jensen TK, Iburg T, Mousing J (1997). Microbiology and pathology of fibrinous pericarditis in Danish slaughter pigs. Zentralbl Veterinarmed A.

[38] Obradovic MR, Segura M, Segalés J, Gottschalk M (2021). Review of the speculative role of co-infections in Streptococcus suis-associated diseases in pigs. Vet Res.

[39] Murase K, Watanabe T, Arai S, Kim H, Tohya M, Ishida-Kuroki K (2019). Characterization of pig saliva as the major natural habitat of Streptococcus suis by analyzing oral, fecal, vaginal, and environmental microbiota. PLoS ONE.

[40] Albernaz-Gonçalves R, Olmos G, Hötzel MJ. Exploring Farmers ’ Reasons for Antibiotic Use and Misuse in Pig Farms in Brazil. antibiotics. 2021;10(331). 10.3390/antibiotics10030331 PMC8004152 33809885

[41] Pagot E, Pommier P, Keïta A (2007). Relationship between growth during the fattening period and lung lesions at slaughter in swine. Rev Med Vet (Toulouse).

